# Prolonged hypothermic machine perfusion enables daytime liver transplantation – an IDEAL stage 2 prospective clinical trial

**DOI:** 10.1016/j.eclinm.2023.102411

**Published:** 2024-01-05

**Authors:** Isabel M.A. Brüggenwirth, Veerle A. Lantinga, Bianca Lascaris, Adam M. Thorne, Mark Meerdink, Ruben H. de Kleine, Hans Blokzijl, Aad P. van den Berg, Koen M.E.M. Reyntjens, Ton Lisman, Robert J. Porte, Vincent E. de Meijer, DHOPE-PRO Trial Investigators, Vincent E. de Meijer, Isabel M.A. Brüggenwirth, Veerle A. Lantinga, Cyril Moers, Diethard Monbaliu, Sijbrand H. Hofker, Jan Bottema, Hildegaard S. Franke, Marieke T. de Boer, Anne Loes van den Boom, Carlijn I. Buis, Suomi M.G. Fouraschen, Frederik J.H. Hoogwater, Vincent E. de Meijer, Joost M. Klaase, Ruben H.J. de Kleine, Mark Meerdink, Maarten W. Nijkamp, Robert J. Porte, A. Michel Rayar, Aad P. van den Berg, Hans Blokzijl, Frans J.C. Cuperus, Frans van der Heide, Frederike G.I. van Vilsteren, Ilhama F. Abbasova, Meine H. Fernhout, Peter Meyer, Ernesto R.R. Muskiet, Koen M.E.M. Reyntjens, Jaap J. Vos, Miriam Zeillemaker, Isabel M.A. Brüggenwirth, Martijn P.D. Haring, Veerle A. Lantinga, Bianca Lascaris, Carol C. Pamplona, Adam M. Thorne, Vivianne Veenma, Otto B. van Leeuwen, Silke B. Bodewes, Ton Lisman, Jelle Adelmeijer, Janneke Wiersema-Buist, Marius van den Heuvel

**Affiliations:** a Department of Surgery, Section of Hepatobiliary Surgery and Liver Transplantation, University of Groningen, University Medical Center Groningen, Groningen, the Netherlands; b UMCG Comprehensive Transplant Center, University Medical Center Groningen, Groningen, the Netherlands; c Department of Gastroenterology and Hepatology, University of Groningen, University Medical Center Groningen, Groningen, the Netherlands; d Department of Anesthesiology, University of Groningen, University Medical Center Groningen, Groningen, the Netherlands; e Surgical Research Laboratory, Department of Surgery, University of Groningen, University Medical Center Groningen, Groningen, the Netherlands

**Keywords:** Liver transplantation, Machine perfusion, Machine preservation, Clinical trial, Hypothermic machine perfusion, IDEAL stage 2

## Abstract

**Background:**

Liver transplantation is traditionally performed around the clock to minimize organ ischemic time. However, the prospect of prolonging preservation times holds the potential to streamline logistics and transform liver transplantation into a semi-elective procedure, reducing the need for nighttime surgeries. Dual hypothermic oxygenated machine perfusion (DHOPE) of donor livers for 1–2 h mitigates ischemia-reperfusion injury and improves transplant outcomes. Preclinical studies have shown that DHOPE can safely extend the preservation of donor livers for up to 24 h.

**Methods:**

We conducted an IDEAL stage 2 prospective clinical trial comparing prolonged (≥4 h) DHOPE to conventional (1–2 h) DHOPE for brain-dead donor livers, enabling transplantation the following morning. Liver allocation to each group was based on donor hepatectomy end times. The primary safety endpoint was a composite of all serious adverse events (SAE) within 30 days after transplantation. The primary feasibility endpoint was defined as the number of patients assigned and successfully receiving a prolonged DHOPE-perfused liver graft. Trial registration at: WHO International Clinical Trial Registry Platform, number NL8740.

**Findings:**

Between November 1, 2020 and July 16, 2022, 24 patients were enrolled. The median preservation time was 14.5 h (interquartile range [IQR], 13.9–15.5) for the prolonged group (n = 12) and 7.9 h (IQR, 7.6–8.6) for the control group (n = 12; p = 0.01). In each group, three patients (25%; 95% CI 3.9–46%, p = 1) experienced a SAE. Markers of ischemia-reperfusion injury and oxidative stress in both perfusate and recipients were consistently low and showed no notable discrepancies between the two groups. All patients assigned to either the prolonged group or control group successfully received a liver graft perfused with either prolonged DHOPE or control DHOPE, respectively.

**Interpretation:**

This first-in-human clinical trial demonstrates the safety and feasibility of DHOPE in prolonging the preservation time of donor livers to enable daytime transplantation. The ability to extend the preservation window to up to 20 h using hypothermic oxygenated machine preservation at a 10 °C temperature has the potential to reshape the landscape of liver transplantation.

**Funding:**

10.13039/501100005075University Medical Center Groningen, the Netherlands.

**Research in context**

**Evidence before this study**

Transplant centers increasingly employ 1–2 h of (dual) hypothermic (10–12 °C) oxygenated machine perfusion (DHOPE) after cold storage to reduce ischemia-reperfusion injury and improve transplant outcomes. Yet, limited evidence exists regarding the safety of extending donor liver preservation using DHOPE for longer durations. In a preclinical study we have successfully preserved donor livers for up to 24 h using DHOPE and in a multicenter observational cohort study we reported excellent transplant outcomes with donor livers preserved by DHOPE for up to 8 h, extending total preservation times to 20 h. Despite these encouraging preclinical and observational findings, according to IDEAL-D (Idea, Development, Exploration, Assessment, Long term study-Framework for Devices), additional evidence is needed to establish the safety and feasibility of prolonged DHOPE before widespread clinical adoption.

**Added value of this study**

In this first-in-human IDEAL-D Stage 2 clinical trial, we demonstrate the safety and feasibility of using DHOPE for prolonging human donor liver preservation for up to 20 h. No significant difference in serious adverse events was observed, and with a minimum one-year follow-up after transplantation patients experienced no clinical or biochemical signs of non-anastomotic biliary strictures, with both graft and patient survival reaching 100% in each group. The ability to safely extend donor liver preservation through DHOPE is poised to revolutionize current liver transplantation practices.

**Implications of all the available evidence**

Prolonged preservation with DHOPE could streamline transplantation logistics and reduce organ discards due to logistical constraints by transforming liver transplantation into a semi-elective procedure, minimizing nighttime surgeries. Daytime liver transplantation could enhance training opportunities for fellows and contribute to a healthier work-life balance for the entire transplant team. To validate and expand on our findings, in our center the transition to daytime liver transplants since January 1st, 2023, is undergoing prospective evaluation through an observational trial (ClinicalTrials.gov; number NCT05680246), representing a radical and unprecedented change in clinical practice.

## Introduction

Liver transplantation is considered an emergency surgical procedure due to the progressive deterioration in the quality of the donor liver with each additional minute of static cold storage (SCS) on ice. The limited window during which donor livers remain viable outside the body currently imposes significant constraints on transplantation. Achieving long-term liver preservation is a crucial element in streamlining transplantation logistics and reducing the number of organs discarded for logistical reasons. Furthermore, extended preservation times could potentially transform liver transplantation into a semi-elective procedure, minimizing the need for nighttime surgeries.

While SCS (∼4 °C) is adequate for short-term preservation of high-quality livers, machine perfusion may be employed as an alternative or adjunct to SCS to prolong preservation time safely. Some centers have implemented normothermic (35–37 °C) machine perfusion (NMP) preservation to reduce the need for nighttime transplantations.^1^ However, the use of machine perfusion at hypothermic temperatures (10–12 °C) to prolong preservation time is not currently widespread, despite its potential benefits, such as reduced costs and lower labor intensity compared to NMP.

During dual hypothermic oxygenated machine perfusion (DHOPE), donor livers are continuously perfused through the portal vein and hepatic artery with a cold, acellular, oxygenated fluid at low vascular pressures.^2^ Within 1 h of DHOPE, mitochondria undergo reprogramming, leading to a subsequent renewal of the nucleotide pool and more effective succinate metabolism.^3^ As a result, reperfusion of livers treated with 1–2 h of DHOPE is associated with reduced ischemia-reperfusion injury and improved outcomes after liver transplantation. Recent randomized clinical trials have reported a significant reduction in early allograft injury, ischemic cholangiopathy, and severe liver-graft related complications when livers were preserved with 1–2 h of (D)HOPE following SCS, compared to SCS alone.^4^, ^5^, ^6^, ^7^

In a preclinical study, we successfully preserved porcine and discarded human livers using prolonged DHOPE for up to 24 h, and these livers exhibited good hepatobiliary function after subsequent warm reperfusion.^8^ Results from a multicenter observational cohort study demonstrated excellent outcomes following transplantation of donor livers preserved by (D)HOPE for up to 8 h, with total preservation times extending to 20 h.^9^ While these findings are promising, additional evidence is required to establish the safety and feasibility of prolonged DHOPE before considering more widespread clinical application, in accordance with the IDEAL-D (Idea, Development, Exploration, Assessment, Long-term study-Framework for Devices) framework.^10^

Here, we present the first-in-human use of prolonged (≥4 h) DHOPE preservation for donation after brain death (DBD) donor livers, aimed at facilitating daytime transplantation. In this investigator-initiated, prospective, dual-arm clinical trial, we assessed the safety and feasibility of prolonged DHOPE compared to the standard (1–2 h) DHOPE method. Our findings indicate no significant differences in the occurrence of serious adverse (device) events (SA[D]E), liver graft-related complications, or patient and graft survival between the two groups. Markers of ischemia-reperfusion injury and oxidative stress measured both in perfusate and in recipients were consistently low and showed no notable discrepancies between the two groups. These results highlight the safety and feasibility of DHOPE as a method to prolong the preservation time for donor livers, thereby enabling successful daytime transplantation. This approach holds the potential to optimize logistical efficiency and further enhance performance in liver transplantation.

## Methods

### Trial design

**Fig. 1:**
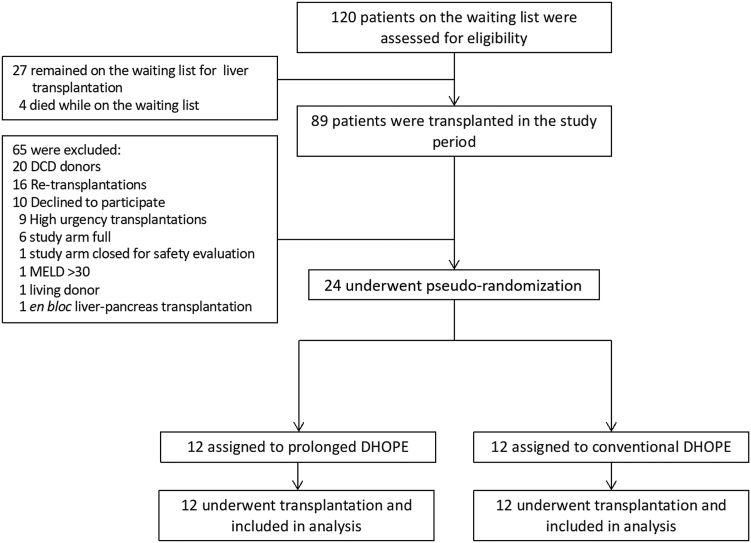
**Consort flow diagram for all recipients enrolled in the trial**. DCD, donation after circulatory death; DHOPE, dual hypothermic oxygenated machine perfusion; MELD, model for end-stage liver disease.

The DHOPE-PRO (Prolonged Dual Hypothermic Oxygenated Machine Perfusion) trial was investigator-initiated and designed as a single-center, prospective, dual arm, pseudo-randomized, clinical safety and feasibility trial (Fig. 1). The trial was conducted at the University Medical Center Groningen (UMCG). IDEAL-D framework and recommendations for Stage 2 clinical device trials were adhered to.^11^^,^^12^ An IDEAL-D Stage 2 trial is designed as a developmental and prospective exploratory study, with the primary objective of assessing safety and feasibility to enable future progression to a definitive randomized controlled trial (Stage 3). The trial protocol was registered at an online repository prior to the initiation of the study (trialregister.nl and the WHO International Clinical Trial Registry Platform; number NL8740) and was previously published.^13^ The protocol was approved by the local Medical Ethical Committee (#2020/126) and the medical-device regulatory body in the Netherlands. The authors designed and implemented the trial, and collected and analyzed the data. The first and last authors drafted the initial manuscript, and all authors contributed to subsequent revisions. All authors affirm the accuracy and completeness of the data and the fidelity of the trial to the protocol.

The independent organ procurement teams were unaware of the trial-group assignment. The donor hepatectomy time was recorded by the independent organ donation coordinator of the organ procurement organization, and communicated with the on-call liver transplant surgeon. Livers were assigned to undergo prolonged DHOPE (≥4 h) if the donor hepatectomy was finished between 16:00 h and 3:59 h (prolonged) and to conventional DHOPE (1–2 h) if the donor hepatectomy was finished between 4:00 h and 15:59 h (control) (Fig. S1). During transportation to the recipient transplant center, livers were preserved by traditional SCS. The trial did not interfere with the regular process of organ allocation or acceptance. All donor livers were matched with a recipient prior to randomization, and no changes in liver recipients occurred after the randomization process.

For logistical reasons, it was not possible to blind the recipient and transplant team. The designated Data Safety Monitoring Board (DSMB) provided adjudication for the primary study endpoint, blinded for treatment assignment.

### Trial patients

Patients 18 years of age or older who were undergoing liver-only transplantation were eligible for inclusion in the trial. The term ‘sex’ is used to describe the sex assigned at birth. Patients were excluded if they were on a high-urgency (HU) status, had a laboratory Model for End-stage Liver Disease (MELD) score >30 or underwent (*en bloc*) combined organ transplantation. Patients receiving a graft from a donation after circulatory death (DCD) donor, a donor with a body weight <40 kg, with an untreated human immunodeficiency virus, or untreated hepatitis B or C virus infection, or with estimated graft steatosis >30% were also excluded. Re-transplantation was an exclusion criterion until the predetermined assessment of the interim results by the independent DSMB after six inclusions in the intervention arm. After the positive evaluation by the DSMB, medical ethical committee approval was obtained for a protocol amendment to start including patients needing re-transplantation (effective by 03/11/2021).

All patients provided written informed consent. The transplantation surgery and postoperative care were performed according to standard local practice.

### Hypothermic oxygenated machine perfusion

The Liver Assist device (XVIVO, Groningen, the Netherlands) was used for *ex situ* end-ischemic DHOPE of the liver. The device enables liver perfusion via both the hepatic artery and the portal vein using two centrifugal pumps to provide a pulsatile and continuous flow, respectively. The system is pressure-controlled, which results in auto-regulation of the flow through the liver. The perfusion pressure was set at 3–5 mmHg for the portal vein and 18–25 mmHg for the hepatic artery. The pressure settings were adjusted to the lowest pressure needed to reach a flow rate of at least 50–200 mL/min in the portal vein and 20–80 mL/min in the hepatic artery. The temperature of the perfusion fluid was maintained at 10 °C. Oxygenation was provided by two membrane oxygenators, each with 100% O_2_ at 500 mL/min to reach partial oxygen pressures of at least 100 kPa. In the intervention group, recipient surgery commenced at around 8:00 h the following morning, and machine perfusion was continued until the recipient hepatectomy was near-finished. Livers in the control group underwent DHOPE for only 1–2 h during recipient hepatectomy.

### End-point measures

The primary safety endpoint was the incidence of SADEs and SAEs during conventional (control) and prolonged (intervention) DHOPE up to 30 days after liver transplantation. This endpoint was defined as the average number of SA(D)Es through the 30 days after liver transplantation per subject. The following SADEs were evaluated: device error, defined as any device error leading to termination of the perfusion; and deviation from the perfusion protocol, defined as any deviation from the perfusion protocol unable to be resolved within 30 min, including temperature >12 °C, oxygenation <100 kPa, pressure >5 mmHg in the portal vein to ensure a flow rate of 50–200 mL/min or a pressure >25 mmHg in the hepatic artery to ensure a flow rate of 20–80 mL/min.

The following SAEs were evaluated: increased hepatic resistance, defined as increased vascular resistance after initiation of machine perfusion (flow <20 mL/min in the hepatic artery or <50 mL/min in the portal vein) in the absence of technical or mechanical issues; post-reperfusion syndrome, assessed according to Aggarwal criteria^14^ or as defined by Watson et al.^15^; primary non-function, defined as non-life sustaining graft function leading to death or re-transplantation within 7 days after liver transplantation; early allograft dysfunction, assessed according to modified Olthoff criteria^16^; vascular complications including a radiologically or surgically proven thrombus of the portal vein and/or hepatic artery; and massive biliary necrosis, defined as the radiological appearance of irregularities and beading dilatation of the intrahepatic bile ducts and/or presence of cavitations and bile lakes leading to surgical or endoscopic intervention within 30 days.

The primary feasibility endpoint was defined as the number of patients assigned and successfully receiving a prolonged DHOPE-perfused liver graft.

Secondary endpoints included biliary complications (including anastomotic and non-anastomotic biliary strictures), 1-year graft and patient survival, the incidence of acute kidney injury, biochemical analysis of graft function and ischemia-reperfusion injury, length of stay at the intensive-care-unit and total hospital length of stay, perfusion characteristics during DHOPE, and major postoperative complications (Clavien-Dindo grade ≥3b) as well as the comprehensive complication index at 30 days.

### Laboratory analysis

The concentration of cell-free DNA (cfDNA) was measured using the Quant-iT PicoGreen double strand DNA assay kit (Fisher Scientific, Landsmeer, the Netherlands). In short, 10 μl of perfusate or plasma was added to 90 μl of Tris–EDTA (TE) buffer (10 mM Tris–HCl, 1 mM EDTA, pH 7.5) in a 96-well microtiter plate. Next, 100 μl of PicoGreen solution (diluted 1:200 in TE buffer) was added to the wells. After 10 min of incubation in the dark at room temperature, fluorescence was measured at 480 nm excitation and 520 nm emission using a Victor3 1420 multilabel counter (PerkinElmer, Groningen, the Netherlands) spectrophotometer.

Thiobarbituric acid reactive substances (TBARS) were measured in perfusate and plasma as a marker for oxidative stress. TBARS were determined using malondialdehyde as a standard as previously described.^17^ D-dimer levels in perfusate and plasma were measured on the StaCompact 3 (Stago, Breda, the Netherlands) using reagents and protocols from the manufacturer.

Hepatic concentration of adenosine-5′-triphosphate (ATP) was used as an indicator of the energy status of the liver grafts. Liver samples were immediately frozen in liquid nitrogen. Frozen tissue was cut into 20 μm slices and a total amount of ±50 mg was homogenized in 1 mL of SONOP (0.372 g EDTA in 130 mL H2O and NaOH (pH 10.9) + 370 mL 96% ethanol) and sonicated. Precipitate was removed by centrifugation (13,000 rcf for 10 min). Supernatant was diluted with SONOP to attain a protein concentration of 200–300 μg/mL (Pierce BCA Protein Assay Kit, Thermo Scientific, Rockford, IL) and mixed with 450 μL of 100 mM phosphate buffer (pH 7.6–8.0). Fifty microliters of phosphate buffered supernatant was used for ATP measurement using ATP Bioluminescence assay kit CLS II (Boehringer, Mannheim, Germany) and a luminometer (Victor3 1420 multilabel counter, PerkinElmer, Groningen, the Netherlands). ATP concentrations were calculated from a calibration curve constructed on the same plate, corrected for amount of protein, and values were expressed as μmol/g protein.

Plasma and perfusate levels of intercellular adhesion molecule 1 (ICAM-1), interleukin 6 (IL-6), tumor necrosis factor alpha (TNF-a), and hyaluronic acid were determined using commercially available enzyme-linked immunosorbent assays (Biotechne, Minneapolis, MN, USA). Levels of high-mobility group box 1 (HMGB-1) were determined by a commercially available enzyme-linked immunosorbent assay from Bioconnect (Huissen, the Netherlands). Tissue plasminogen activator (tPA) levels were determined using a Zymutest kit from Nodia (Amstelveen, the Netherlands).

### Immunohistochemistry

Liver parenchyma biopsies were fixated in 10% buffered formalin immediately after collection. The fixated biopsies were then embedded in paraffin and cut into 3 μm sections. Standard protocols were used to process the sections for hematoxylin and eosin (HE) staining. For immunohistochemical von Willebrand Factor (VWF) and Caspase-3 staining, endogenous peroxidase activity was blocked by incubating the sections in 3% hydrogen peroxide for 30 min. Antigen retrieval was induced by applying 0.2% pepsin pH 2 for VWF and heat-induced with EDTA pH 8.0 for Caspase-3. Sections were then incubated for 60 min at room temperature with the primary antibody (antigen VWF, rabbit host, supplier Dako Cytomation, cat no. A0082; antigen Caspase-3, rabbit host, supplier Cell Signaling Technology, cat no. 9661) diluted to 1:250 for VWF and to 1:100 for Caspase-3. Visualization was achieved by peroxidase-labeled goat–anti-rabbit and rabbit–anti-goat antibodies (DAKO, Glostrup, Denmark), diluted to 1:100 for VWF and to 1:50 for Caspase-3. Subsequently, a diaminobenzidine substrate was added, followed by counterstaining with hematoxylin.

### Safety and trial oversight

A pre-specified case-by-case analysis was performed by the designated DSMB for the first six subjects in the intervention group. These patients were evaluated up to two weeks after liver transplantation, with the inclusions for the intervention group being temporarily halted during that period. After including 6 patients in the intervention group, an interim analysis revealed equally low rates of SA(D)Es in the intervention group, which allowed us (as prespecified per protocol) to discard the two-week evaluation period after each new case in the intervention group.

The study was initially designed with stratification for DBD (12 vs. 12) and DCD (6 vs. 6) livers.^13^ However, due to the impact of the COVID-19 pandemic and the resulting strain on intensive care unit beds, DCD liver procedures in the Netherlands were limited to daytime scheduling, imposing logistical constraints. As a result, we were unable to include livers in the experimental group for the DCD arm. Following formal written permission from the ethical committee, we made the decision to limit the study exclusively to DBD-only cases, which represent over 90% of all liver transplants worldwide. Therefore, the total number of patients included in this trial is 24, with 12 DBD liver recipients in the control arm and 12 DBD liver recipients in the experimental arm.

### Statistical analysis

All endpoint analyses were prespecified in the protocol. The primary safety endpoint, a composite of the incidence of SADEs and SAEs, is presented as actual numbers and mean. The primary feasibility endpoint, the risk of patients successfully receiving the intervention, is presented as actual numbers and mean. The secondary endpoints are presented as actual numbers and mean. For each group, we calculated a 95% confidence interval (CI) for the mean using the t-distribution. These calculations were based on the assumption that the number of events followed a normal distribution and that the sample was representative of the population. Proportions for both the primary and secondary endpoints were compared using a Fisher's exact test. Fixed sequence testing was used for the secondary endpoints, eliminating the need for adjustment for multiplicity. The secondary endpoints were tested in the order as presented above. Continuous data are presented as mean ± standard deviation when normally distributed, or as median and IQR. Statistical analysis was performed using IBM SPSS Statistics version 27 and GraphPad Prism 2022, Version 9.4.1.

We adhered to the CONSORT checklist when writing our report.^18^

### Role of the funding source

The funder of the study had no role in study design, data collection, data analysis, data interpretation, or writing of the report.

## Results

### Recruitment

Between November 1, 2020 and July 16, 2022, we assessed 120 consecutive adult patients for eligibility, of whom 89 were transplanted during the study period. Of those, 24 patients underwent randomization and transplantation (Fig. 1). Notably, the trial was initiated and recruitment was completed during the COVID-19 pandemic (Fig. S2).

### Donor, recipient, and preservation characteristics

**Fig. 2:**
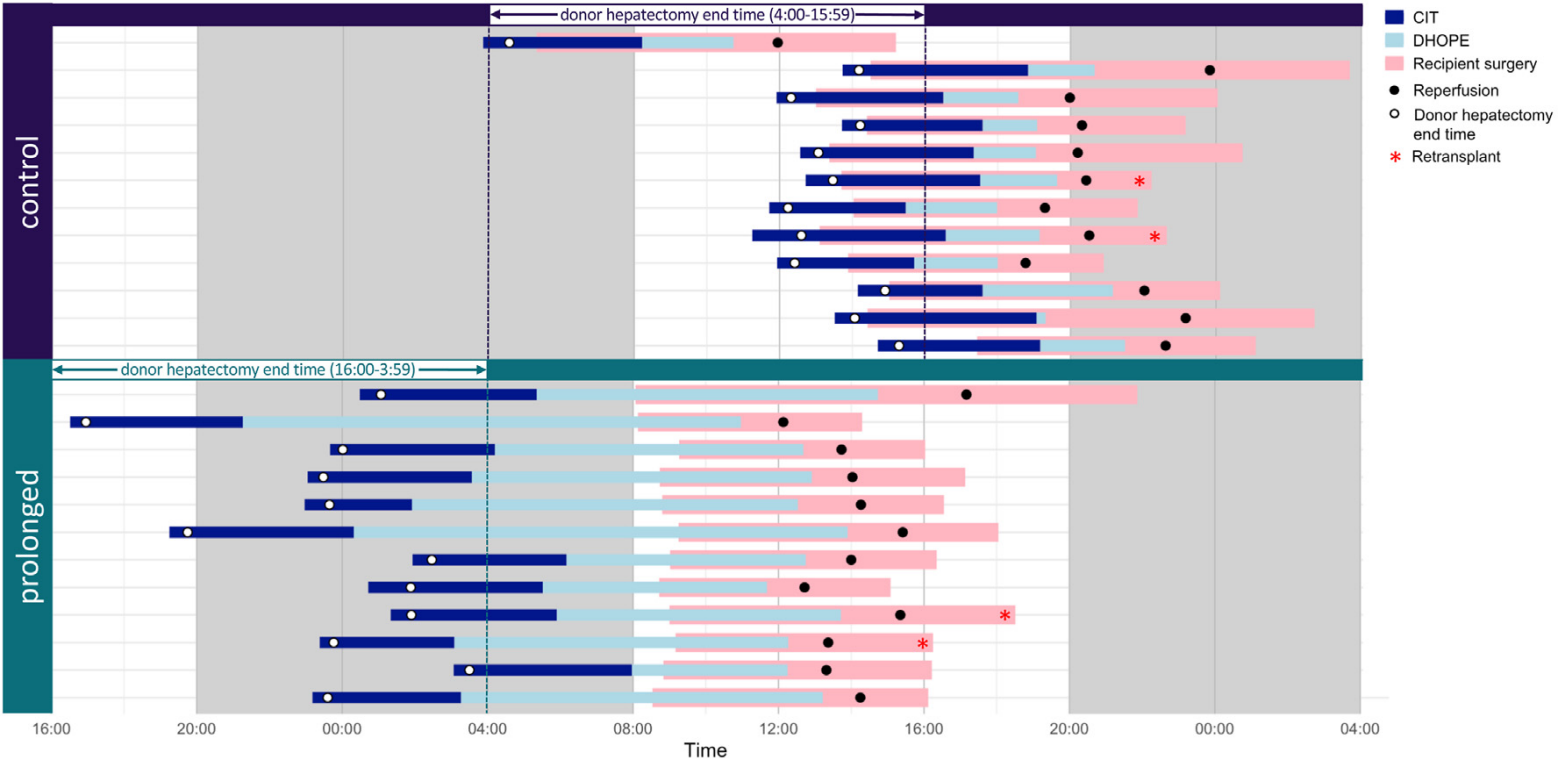
**Cold ischemia, preservation, and recipient transplant surgery times according to the treatment group**. Cold ischemia time (CIT), dual hypothermic oxygenated machine perfusion (DHOPE) preservation time, and recipient transplant surgery duration is plotted for each individual procedure for the control group (*n* = 12), and for the prolonged preservation intervention group (*n* = 12). Donor hepatectomy end time (which determined the assignment to each study arm) as well as the recipient reperfusion time is indicated for each case. Two liver retransplantations in each group are indicated with an asterisk.

**Fig. 3:**
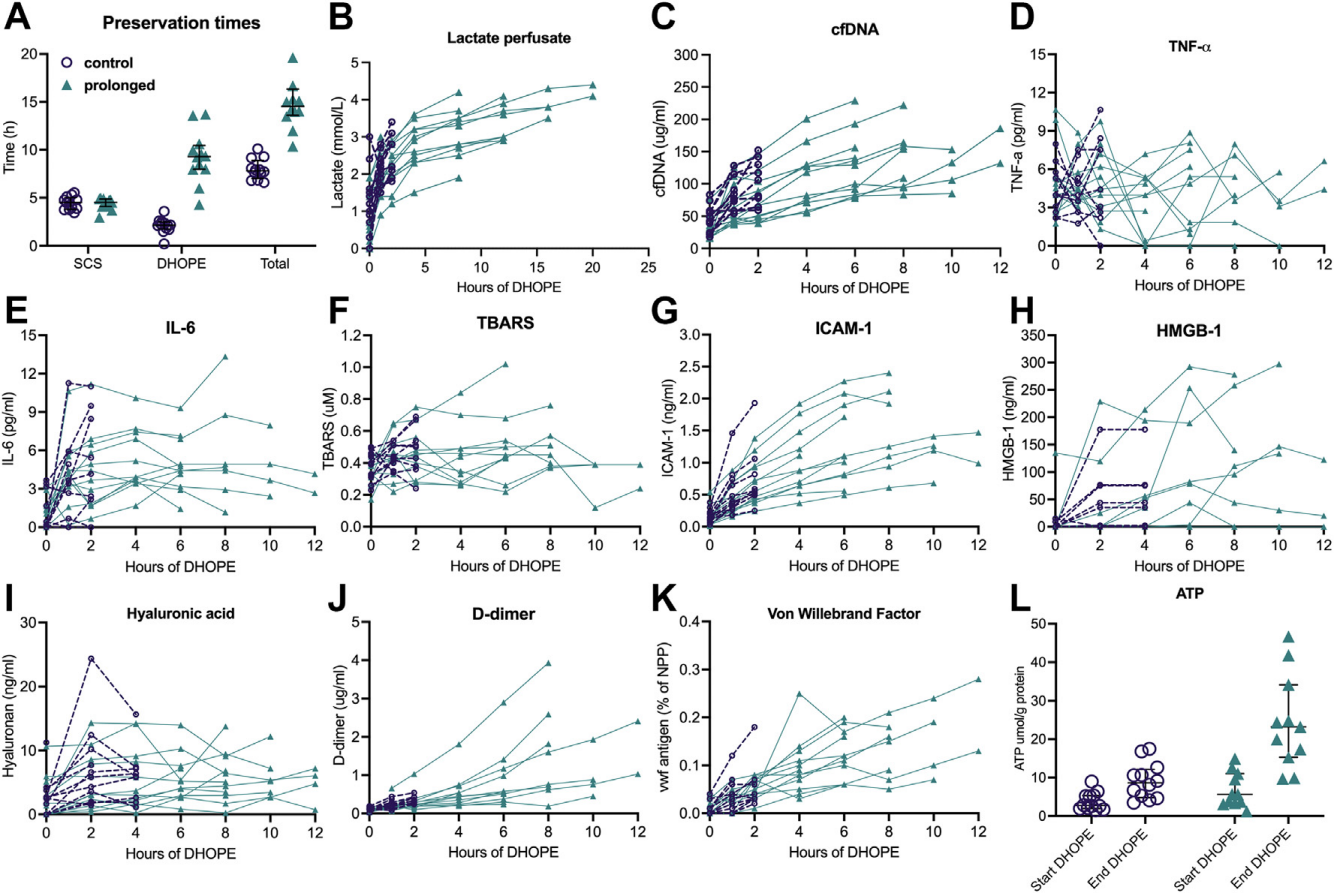
**Preservation times and perfusate markers duringdual hypothermic oxygenatedmachine perfusion(DHOPE)according to the treatment group**. Preservation times (A) are plotted individually for the control group (*n* = 12), and for the prolonged preservation intervention group (*n* = 12). The middle line is the median, the lower and upper whisker represent the first and third quartiles. Individual values during perfusion are presented for lactate (B), cell-free DNA (cfDNA; C), tumor necrosis factor alpha (TNF-a; D), interleukin 6 (IL-6; E), thiobarbituric acid reactive substances (TBARS; F), intercellular adhesion molecule 1 (ICAM-1; G), high-mobility group box 1 (HMGB-1; H), hyaluronic acid (I), D-dimer (J), and von Willebrand Factor (K). Individual levels of adenosine triphosphate (ATP) of liver biopsies at baseline (before start DHOPE) and at the end of perfusion (end DHOPE) (L).

The baseline characteristics of the donors, recipients, and static cold preservation time during transport to the recipient hospital were similar in the two trial groups (Table 1). Within each group, two liver retransplantations (17%) were performed. Of these, in the control group, one patient required a second liver transplant after 11 months due to ischemic cholangiopathy. Additionally, three other patients, one from the control group and two from the prolonged DHOPE group, underwent retransplantation due to recurrent primary sclerosing cholangitis after 7.5 years, 6.5 years, and 20 years, respectively. In the control group, all liver transplants except one commenced before 16:00 h. In contrast, in the prolonged DHOPE group, nearly all surgeries began between 8:00 and 9:00 h, with 10 out of 12 surgeries completed before 18:00 h (Fig. 2). Notably, median recipient transplant surgery duration in the prolonged group (7.5 h; interquartile range [IQR], 6.9–8.7) was almost 2 h shorter, compared to the control group (9.3 h; IQR, 8.0–11.3; p = 0.02). Inherent to the intervention, the median machine perfusion time was longer for the prolonged DHOPE group (9.3 h; IQR, 8.0–10.1), compared to the control group (2.2 h; IQR, 2.0–2.4). Likewise, the median total preservation time was longer for the prolonged group (14.5 h; IQR, 14.0–15.6), compared to the control group (7.9 h; IQR, 7.6–8.6) (Fig. 3A).

**Table 1:** Donor, recipient, and preservation characteristics.

	Control (*n* = 12)	Prolonged (*n* = 12)	p-value
**Donor characteristics**			
Age (y)	51 ± 14	49 ± 18	0.74
Female sex (no.)	6	5	1
Body mass index (kg/m^2^)	28 ± 5	25 ± 4	0.07
Cause of death (no.)			0.79
Cerebrovascular accident	7	8	
Anoxia	2	1	
Trauma	1	2	
Other	2	1	
Donor risk index^a^	1.63 (1.48–1.71)	1.69 (1.45–1.92)	0.64
Eurotransplant donor risk index^b^	1.61 (1.44–1.69)	1.70 (1.30–1.93)	0.96
**Recipient characteristics**			
Age (y)	54 ± 12	47 ± 18	0.30
Female sex (no.)	6	5	1
Indication for transplantation (no.)			0.32
Re-transplantation	2	2	
Primary sclerosing cholangitis	2	4	
Non-alcoholic steatohepatitis	2	2	
Hepatocellular carcinoma	0	2	
Polycystic liver disease	3	0	
Other	3	2	
Laboratory MELD score	15 (12–20)	18 (12–22)	0.60
Balance of risk score^c^	5 (4–9)	7 (4–10)	0.76
Preservation characteristics			
Static cold storage time^d^ (hours)	4.5 (3.8–4.9)	4.5 (4.1–4.8)	0.94
Machine perfusion duration (hours)	2.2 (2.0–2.4)	9.3 (8.0–10.1)	0.01
Warm ischemia time (minutes)	34 (31–38)	34 (30–36)	0.62
Total preservation time^e^ (hours)	7.9 (7.6–8.6)	14.5 (13.9–15.6)	0.01

Data are presented as actual numbers, as mean ± standard deviation when normally distributed, or as median (interquartile range).

a The donor risk index was developed by Feng et al. as a scoring system that was developed to quantitatively predict the risk of post-transplantation graft failure in liver transplantation.

b The Eurotransplant donor risk index was developed by Braat et al. as a modification on the donor risk index specifically for the Eurotransplant region.

c The balance of risk score was developed by Dutkowski et al. to detect unfavorable combinations of donor and recipient factors on post-transplant survival.

d Static cold storage time was defined as the time from cold perfusion in the donor until the start of machine perfusion.

e Total preservation time was defined as the time from cold perfusion in the donor until reperfusion in the recipient.

### Primary safety and feasibility endpoint

For the primary safety endpoint, in each group three patients (25%; 95% CI 3.9–46%, p = 1) developed a SA(D)E within the first 30 days after transplantation. One device error (SADE) occurred in the control group. During machine perfusion, the pressure sensor malfunctioned, leading to inaccurate pressure measurements and unreliable flows. This liver was disconnected from the perfusion device 5 min after the initiation of machine perfusion. The graft was transplanted with an uneventful postoperative course. No SADEs occurred in the prolonged group. Post-reperfusion syndrome occurred in three patients (25%; 95% CI 3.9–46%) in the prolonged group and in two recipients (17%; 95% CI –2.3 to 36%) in the control group (Table 2). Other predefined liver graft- or device-related SAEs, including increased hepatic resistance, primary non-function, early allograft dysfunction, vascular complications, or massive biliary necrosis, did not occur in any group.

**Table 2:** Patient outcomes.

	Control (*n* = 12)	Prolonged (*n* = 12)
**Primary safety outcome**		
Any serious adverse (device) event (no.)	3	3
Serious adverse device event (no.)		
Device error	1	0
Deviation from the perfusion protocol	0	0
Serious adverse event (no.)		
Increased hepatic resistance	0	0
Post-reperfusion syndrome^a^	2	3
Primary non-function	0	0
Early allograft dysfunction	0	0
Vascular complications	0	0
Massive biliary necrosis	0	0
**Primary feasibility outcome**		
Successful prolonged perfusion (no.)	N/A	12
**Secondary outcomes**		
Biliary complications		
Anastomotic strictures (no.)	3	3
Non-anastomotic strictures (no.)	0	0
Actual 1-year survival		
Graft	100%	100%
Patient	100%	100%
Acute kidney injury (no.)	2	3
Requiring CVVH (no.)	1	0
Length of stay		
Intensive care (days)	1 (1–2)	1 (1–1)
Total (days)	11 (10–17)	10 (8–12)
Postoperative complications		
Clavien-Dindo grade ≥3B (no.)	5	4
Comprehensive complication index	10.5 (8.7–37.4)	17.4 (2.2–43.2)

Data are presented as actual numbers or as median (interquartile range).

a According to the definition of Aggarwal et al. (a decrease in mean arterial pressure >30% below baseline, lasting for ≥1 min, within 5 min after reperfusion) and Watson et al. (vasoplegia with a fall in mean arterial pressure on reperfusion to <50 mmHg either sustained >30 min and/or requiring >0.15 μg/kg/min norepinephrine, >2 U/hour vasopressin, or infusion of epinephrine). In the prolonged group, 1/3 recipients met the definition of post-reperfusion syndrome according to Aggarwal et al. and 2/3 recipients according to Watson et al. In the control group, both recipients met de definition according to Watson et al.

For the primary feasibility endpoint, all patients assigned to either the prolonged group or control group successfully received a liver graft perfused with either prolonged DHOPE or control DHOPE, respectively.

### Secondary endpoints

#### Postoperative complications

Prespecified secondary endpoints are summarized in Table 2. After a median follow-up time of 19 months (range 12–32 months; IQR 16–27 months), 3 patients in each group (25%; 95% CI 3.9–46%, p = 1) were diagnosed with a biliary anastomotic stricture (AS). With a minimum follow-up of at least 1 year after transplantation, none of the grafts showed clinical, nor biochemical signs of non-anastomotic biliary strictures. Graft and patient survival are 100% at 1 year after liver transplantation in both groups. Acute kidney injury occurred in three patients in the prolonged group vs. two patients in the control group, of which one recipient in the latter group required hemodialysis. Patients in the prolonged group had a median length of stay in the intensive care unit of 1 day (IQR, 1–1) and a median total hospital length of stay of 10 days (IQR, 8–12). Patients in the control group had a median length of stay in the intensive care unit of 1 day (IQR, 1–2) and a median total hospital length of stay of 11 days (IQR, 10–17). In the prolonged group, four patients experienced postoperative complications Clavien-Dindo grade 3B or higher. These included two patients with an AS, one of whom required an endoscopic retrograde cholangiopancreatography (ERCP) with stenting, and another whom required percutaneous transhepatic drain (PTCD) placement under general anesthesia. Two other patients required a relaparotomy for postoperative bleeding. In the control group, five patients experienced postoperative complications Clavien-Dindo grade 3B or higher. Two patients had an AS and required ERCP with stenting. A third patient required PTCD placement under general anesthesia for an AS. Another patient experienced acute kidney injury and required hemodialysis for 3 days. A fifth patient required a relaparotomy for postoperative bleeding. The median comprehensive complication index at 30 days was 17.4 (IQR, 2.2–43.2) in the prolonged vs. 10.5 (IQR, 8.7–37.4) in the control group.

#### Machine perfusion data

During DHOPE, resistance in the hepatic artery and portal vein remained stable (Fig. S3). Perfusate temperatures were maintained <12 °C throughout the perfusion. Perfusate levels of ischemia-reperfusion injury markers (lactate, cfDNA, TNF-α, and IL-6) increased only marginally during prolonged DHOPE (Fig. 3B–E). Perfusate levels of oxidative stress markers (TBARS, ICAM-1, and HMGB-1) did not further increase during prolonged perfusion (Fig. 3F–H). As a marker for endothelial injury, hyaluronic acid levels did not increase, while we observed increasing levels of D-dimer and VWF during prolonged perfusion (Fig. 3I–K). ATP levels in liver biopsies were higher at the end of prolonged DHOPE compared to controls with a median of 23.2 μmol/g protein vs. 8.6 μmol/g protein, respectively (Fig. 3L).

#### Graft function and injury after transplantation

**Fig. 4:**
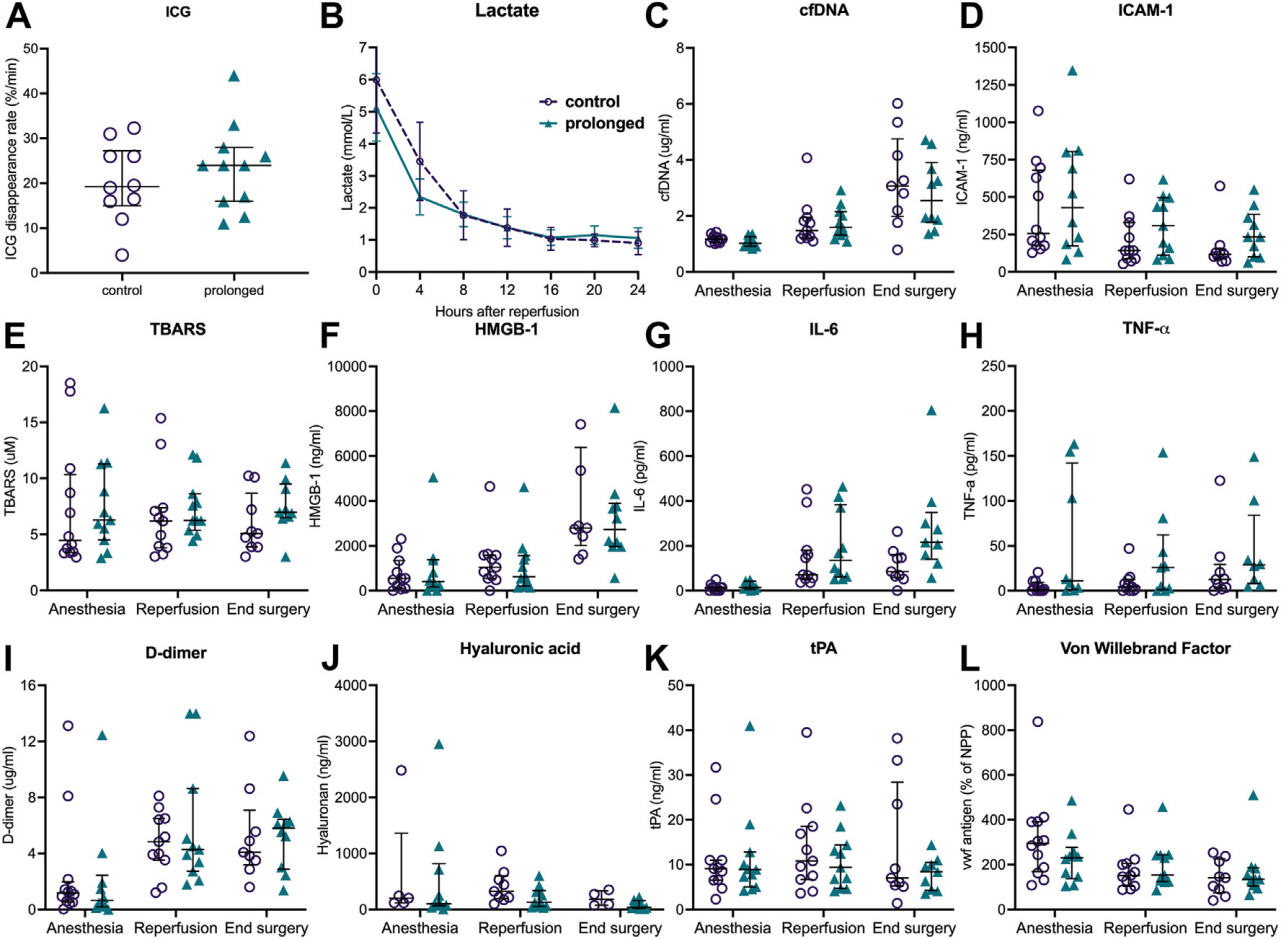
**Serum markers before and after reperfusion in the recipient according to the treatment group**. Indocyanine green (ICG) plasma disappearance rates after reperfusion (A) are plotted individually for the control group (n = 12), and for the prolonged preservation intervention group (n = 12). The middle line is the median, the lower and upper whisker represent the first and third quartiles. Lactate clearance in the recipient during the first 24 h after reperfusion with median and 95% confidence interval (B). Individual values before surgery (anesthesia), 5 min after reperfusion, and at end of surgery are presented for cell-free DNA (cfDNA; C), intercellular adhesion molecule 1 (ICAM-1; D), thiobarbituric acid reactive substances (TBARS; E), high-mobility group box 1 (HMGB-1; F), interleukin 6 (IL-6; G), tumor necrosis factor alpha (TNF-a; H), D-dimer (I), hyaluronic acid (J), tissue plasminogen activator (tPA; K), and von Willebrand Factor (L).

Indocyanine green plasma disappearance rate measured after graft reperfusion was similar in both groups, with a median of 24.0%/min (IQR, 16.0–28.0) in the prolonged group and 19.3%/min (IQR, 15.0–27.3) in the control group (Fig. 4A). After reperfusion in the recipient, serum markers of ischemia-reperfusion injury, oxidative stress and endothelial injury were not different between the groups when measured 5 min after reperfusion and at the end of surgery (Fig. 4B–L). Median serum IL-6 levels, however, were somewhat higher in the prolonged group (216 pg/mL; IQR, 140–348) compared to the control group (85 pg/mL; IQR, 58–161) at the end of surgery (Fig. 4G).

**Fig. 5:**
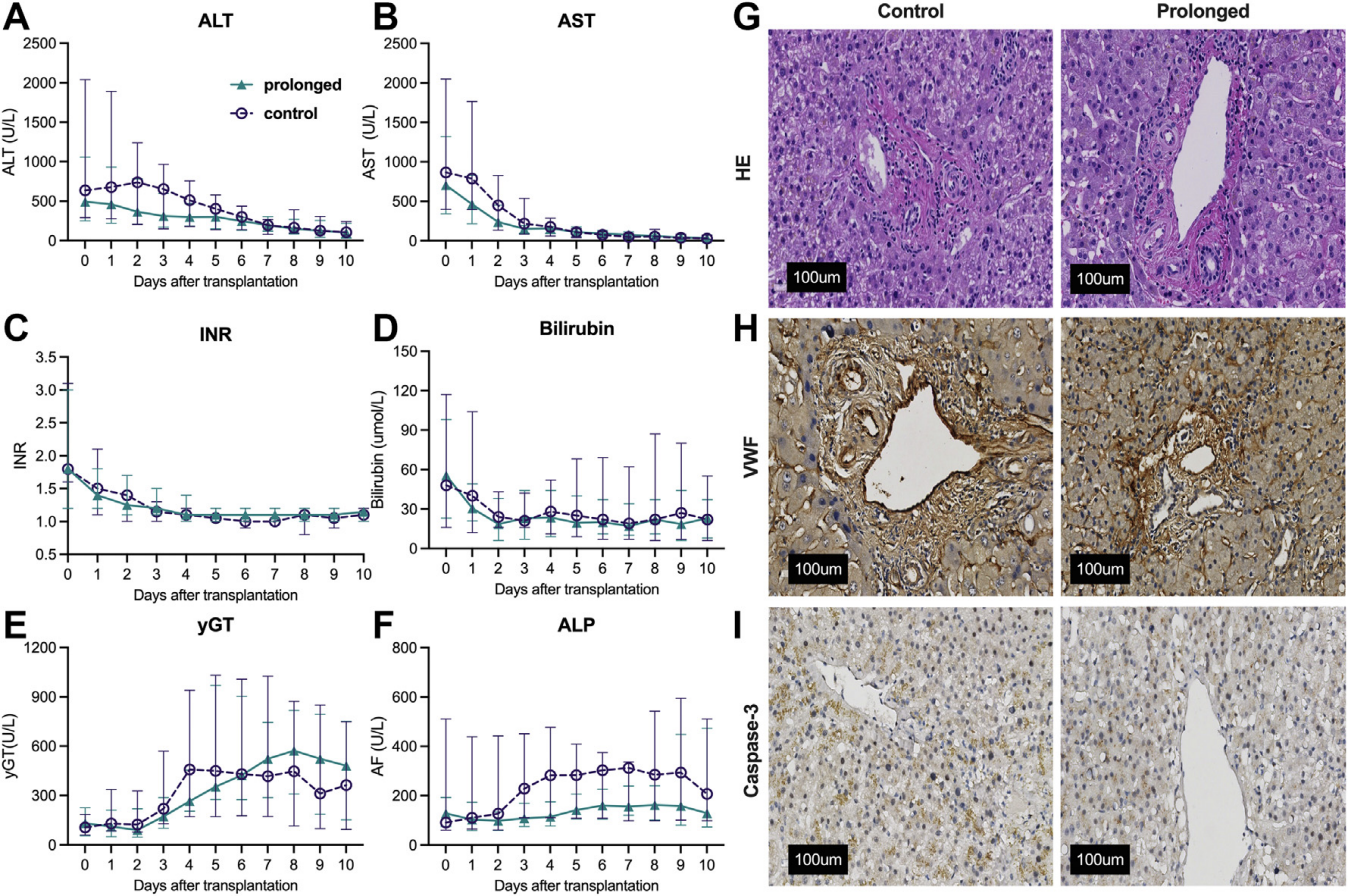
**Postoperative biochemistry after transplantation in the recipient and histological analysis of liver biopsies after reperfusion according to the treatment group**. Values for alanine aminotransferase (ALT; A), aspartate aminotransferase (AST; B), international normalized ratio (INR; C), total bilirubin (D), gamma glutamyl transferase (yGT; E), and alkaline phosphatase (ALP; F) are plotted individually for the control group (n = 12), and for the prolonged preservation intervention group (n = 12) up to 10 days after transplantation. The middle line is the median, the lower and upper whisker represent the 95% confidence interval. Representative images from histological assessment of liver biopsies after reperfusion in the recipient are depicted for the control group and the prolonged preservation group (G–I). Hematoxylin and eosin (HE) stain showed some subcapsular neutrophils and minimal hepatocellular injury in controls as well as in the prolonged group (G). Von Willebrand Factor (VWF) staining showed no significant vascular endothelial cell activation in control (H) or prolonged perfusion groups. Caspase-3 staining did not reveal apoptosis in any of the groups (I).

After transplantation, median peak serum ALT was 492 U/L (IQR, 259–1026) in the prolonged group and 738 U/L (IQR, 274–1101) in the control group (Fig. 5A). Other biochemistry during the first 10 postoperative days was not different between the groups for serum AST (Fig. 5B), international normalized ratio (INR; Fig. 5C), total bilirubin (Fig. 5D), and gamma glutamyl transferase (yGT) (Fig. 5E). Median levels for alkaline phosphatase (ALP) on postoperative days 4–6 were higher in the control group (peak day 6, 346 U/L; IQR, 169–303), when compared to the prolonged group (peak day 6, 225 U/L; IQR, 105–161) (Fig. 5F).

#### Histology

Histological analysis of liver parenchyma biopsies before the start of machine perfusion revealed no pre-existing histological abnormalities (*data not shown*). In the biopsies taken after reperfusion in the recipient, HE staining showed intact architecture of the liver. The presence of neutrophils in the portal triads was slightly more abundant, indicating some ‘surgical hepatitis’. This nonpathological phenomenon induced by surgical manipulation was not more severe in either of the groups (Fig. 5G). VWF-stained sections showed intact endothelial linings of the central veins, sinusoids and portal tracts. The staining intensity was similar for both groups, indicating no differences in endothelial activation (Fig. 5H). Caspase-3 was not abundant in biopsies after reperfusion. Zone 3 hepatocytes, where most apoptosis induced by oxidative stress could be expected, also appeared vital in both groups (Fig. 5I).

## Discussion

Liver transplant logistics are currently constrained by the length of time the organ can be kept viable upon removal from the donor. This first-in-human clinical trial demonstrates the safety and feasibility of prolonged preservation of donor livers with DHOPE for up to 20 h, enabling successful daytime transplantation without an increased rate of predefined serious adverse events.

No SADEs occurred during prolonged DHOPE, and perfusion parameters remained stable over time. Throughout all (prolonged) liver perfusions, the liver's temperature was maintained between 10 and 12 °C, and half-hourly gas analyses consistently indicated a partial oxygen pressure of the perfusion fluid exceeding 100 kPa at all time points. Intrahepatic vascular resistance in both the portal vein and hepatic artery remained stable during perfusion. While, during this trial, at least one organ perfusionist was present at all times to monitor the perfusion process, the evident stability of perfusion parameters could enable remote monitoring in the future.

The safety of prolonged DHOPE is further demonstrated by an equally low number of SAEs after liver transplantation, compared to conventional DHOPE. All livers showed immediate graft function with low transaminase levels after transplantation, a rapid recovery of INR, and decreasing bilirubin levels. Early allograft dysfunction was not observed in any group. Both one-year graft and patient survival rates were 100%, and after a minimum follow-up of 12 months none of the recipients have developed non-anastomotic ischemic cholangiopathy.

ATP levels measured in liver tissue at the end of machine perfusion were higher after prolonged DHOPE than after conventional DHOPE. Previous studies have demonstrated that 1–2 h of DHOPE are sufficient to restore cellular ATP levels.^5^^,^^6^^,^^19^ The current data shows that prolonging DHOPE for up to 14 h further increases ATP levels, supporting our hypothesis that this enhanced ATP availability aids hepatic cells in regaining function after reperfusion in the recipient.

During prolonged DHOPE, markers of ischemia-reperfusion injury and oxidative stress did not show significant elevations compared to conventional DHOPE. While these findings are reassuring, it is important to acknowledge that there may be other injury biomarkers not measured in our study that could potentially exhibit different patterns. Therefore, future studies on prolonged DHOPE should consider these aspects to achieve a comprehensive understanding of the effects of this technique.

On the other hand, during prolonged DHOPE, we observed increasing levels of hemostatic proteins such as D-dimer and VWF. Increasing levels of D-dimer in the perfusate probably indicate ongoing, low-grade degradation of micro-thrombi, which we postulate are better washed out during prolonged perfusion.^20^ Increasing perfusate levels of VWF, albeit at a very low level, could potentially be attributed to ongoing, low-grade endothelial cell activation or injury. However, VWF is also constitutively released, and thus, the increase in VWF could suggest low-grade VWF synthesis with subsequent release, despite the low temperature. Supporting this latter mechanism, perfusate levels of hyaluronic acid, another marker for endothelial cell activation, remained stable throughout perfusion. Additionally, VWF staining intensity was similar in both groups, indicating no differences in endothelial activation.

The safe extension of *ex situ* preservation time for donor livers carries significant clinical implications. Currently, the limited time available for donor organ allocation, transportation, and transplantation results in the discard of organs. Even good-quality donor livers can be declined by transplant centers during the allocation procedure, sometimes necessitating extended allocation or rescue allocation procedures, or even leading to organ discard. For instance, in the United States, liver discard rates were higher during public holidays compared to non-holidays.^21^ Prolonged DHOPE can improve logistical efficiency, allowing for the optimal utilization of deceased donor livers while maintaining favorable outcomes.

In this trial, extending the preservation time of liver grafts with DHOPE enabled the scheduling of all transplantations during the day when a greater number of highly skilled personnel is available for complex and high-risk cases, such as re-transplantations. Transforming liver transplantation from an urgent to a semi-elective, daytime surgery, may help mitigate the additional risks associated with nighttime surgery.^22^ Additionally, inadequate sleep and circadian rhythm disruption for the attending transplant team should not be underestimated and can be avoided.^23^ Epidemiological studies have linked circadian clock disruption to an increased risk of several types of cancer, metabolic syndrome, a higher body mass index, insulin resistance, and ageing-related diseases such as Alzheimer's disease.^23^^,^^24^ Furthermore, prolonged DHOPE can be used to simultaneously accept two livers and facilitate sequential transplantation, to accomodate sequential combined thoraco-abdominal transplantation (e.g., heart-liver or lung-liver), or to postpone transplant surgery when an operating room or intensive care bed is not immediately available. Lastly, prolonged perfusion may also enable a split liver procedure on the pump without time pressure or extending ischemia time.^25^

Prolonged preservation at hypothermic temperatures offers several advantages over previously described preservation techniques such as NMP or subzero preservation. Transplantation of donor livers after prolonged NMP up to 24 h has been reported previously.^26^ In a preclinical study, NMP enabled the preservation of porcine and discarded human livers for up to 1 week.^27^ However, during NMP, the liver is metabolically active and produces waste products such as urea and phosphate,^28^ which are recirculated in the perfusion solution and may reach supra-physiological levels.

In contrast, DHOPE preservation maintains the organ at around 10 °C in a hypometabolic state with low vascular pressures. This approach enhances safety and simplicity, reducing labor requirements and costs compared to NMP. Researchers have employed a combined protocol involving sub-normothermic (at 21 °C), hypothermic (at 4 °C), and subzero (at −4 °C) preservation to preserve discarded human livers up to 27 h.^29^ However, subzero preservation of donor organs remains labor-intensive and is still in a preclinical experimental phase.

One of the current limitations of DHOPE, in general, is the inability to evaluate hepatobiliary function during perfusion prior to transplantation. Flavin mononucleotide in the perfusion solution has been proposed as a potential marker for graft function after transplantation. However, more clinical evidence is required to establish flavin mononucleotide as a reliable biomarker of hepatobiliary viability.^3^ Bile production ceases under hypothermic conditions, further complicating the assessment of bile duct viability. In the future, sequential application of prolonged DHOPE preservation followed by NMP could leverage the benefits of resuscitation and subsequent viability assessment for high-risk donor livers.^30^

We acknowledge the careful selection of donor livers and recipients in the current first-in-human prospective clinical trial. This deliberate choice aimed to prioritize the primary objective of assessing the safety of DHOPE in prolonging preservation time. Consequently, the safety and efficacy of prolonged DHOPE beyond our inclusion criteria remain to be established. A limitation of this study is its unblinded nature and the absence of formal randomization, which may potentially introduce residual bias, including confounding. Nevertheless, assignment to each study arm was determined based on the end-time of the donor hepatectomy, which, in all cases, was performed by an independent organ retrieval team unrelated to the recipient transplant team. As a result, all transplants during the study took place during the daytime, enhancing the comparability of transplantation circumstances in each arm.

To conform to and expand upon the results of the current Stage 2 trial, according to the IDEAL-D framework, future research should encompass large-scale, multicentre, prospective studies at the assessment (Stage 3) and long-term study (Stage 4) stages. In our center, we have transitioned virtually all liver transplants to daytime procedures since January 1st, 2023. This radical and unprecedented change in practice is currently under prospective investigation in an observational trial (ClinicalTrials.gov, number NCT05680246).

In conclusion, this pioneering clinical trial demonstrates the safety and feasibility of DHOPE in prolonging the preservation time of donor livers to enable daytime transplantation. The ability to extend the preservation window to up to 20 h using hypothermic oxygenated machine preservation at a 10 °C temperature has the potential to reshape the landscape of liver transplantation.

## Contributors

Vincent E. de Meijer is the principal investigator and guarantor of this article. He was involved in study design and conceptualization, methodology, performed the research, data assessment and verification, project administration, supervision, writing of the original draft and review.

Isabel M.A. Brüggenwirth was involved in study design and conceptualization, methodology, performed the research, data assessment and verification, project administration, and writing of the original draft.

Veerle A. Lantinga was involved in study design and conceptualization, methodology, performed the research, data assessment and verification, and writing of the original draft.

Bianca Lascaris, Adam M. Thorne, Mark Meerdink, Ruben H. de Kleine, Hans Blokzijl, Aad P. van den Berg, and Koen M.E.M. Reyntjens performed the research.

Ton Lisman was involved in methodology and performed the research.

Robert J. Porte was involved in study design and conceptualization, methodology, and performed the research.

All authors critically reviewed the manuscript, confirmed the completeness and accuracy of the report, and agreed on its submission for publication.

Vincent E. de Meijer, Isabel M.A. Brüggenwirth, and Veerle A. Lantinga accessed and verified the data reported.

The study was performed in collaboration with the DHOPE-PRO Trial Investigators (listed in the Supplementary Appendix).

## Data sharing statement

All data supporting the findings of this study are available within the paper and its Supplementary Information. The individual patient data that support the findings of this study are not openly available due to legal and ethical restrictions associated with patient confidentiality. However, anonymized data can be made available from the corresponding author upon reasonable request.

## Declaration of interests

Vincent E. de Meijer reports a VENI research grant by the Dutch Research Council (NWO; grant #09150161810030), a Research grant from the Dutch Ministry of Economic Affairs (Health ∼ Holland Public Private Partnership grant #PPP-2019-024), and a Research grant from the Dutch Society for Gastroenterology (NVGE #01-2021), all outside the submitted work. Other authors declare that they have no competing interests.

## Appendix A Supplementary data

Supplementary Material

Study protocol

List of DHOPE-PRO Trial Investigators
