# Supplementary material for: Prolonged hypothermic machine perfusion enables daytime liver transplantation – an IDEAL stage 2 prospective clinical trial
Source: eClinicalMedicine. 2024 Jan 5;68:102411. doi: 10.1016/j.eclinm.2023.102411 (PMC10789636; doi:10.1016/j.eclinm.2023.102411)
Supplement: Supplementary Material [file mmc1.docx]

**Supplementary data**

**Table of contents**

| **Item** | **Page** |
| --- | --- |
| Figure S1. Graphic representation of the randomization process and trial-group assignment, including the pre-specified primary and secondary endpoints. | 2 |
| Figure S2. Expected versus actual inclusions during the trial study period. | 3 |
| Figure S3. Median perfusion resistance (pressure/flow) of the hepatic artery (HA; left panel) and portal vein (PV; right panel) during dual hypothermic oxygenated machine perfusion (DHOPE) for the control group (n=12), and for the prolonged preservation group (n=12). | 4 |
| List of DHOPE-PRO Trial Investigators | 5 |

**
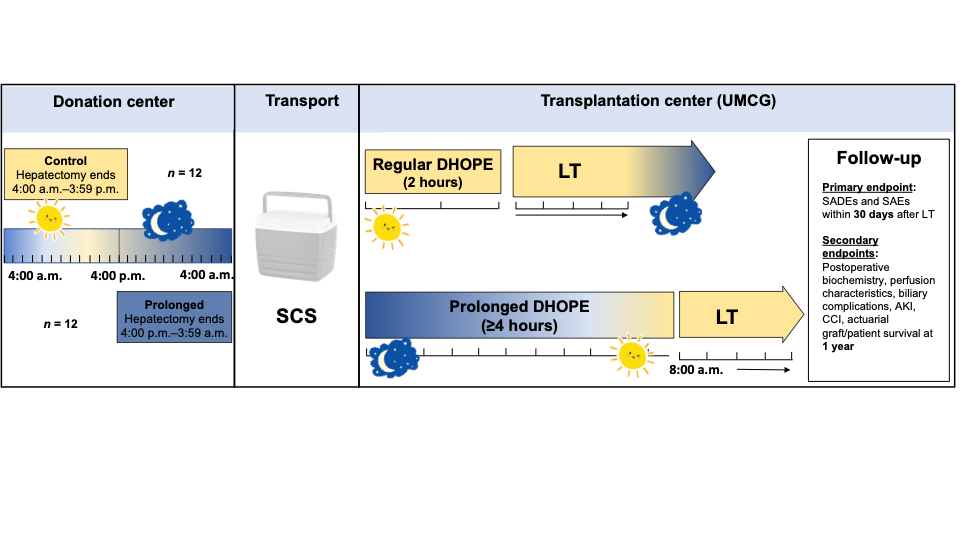
**

**Figure S1. Graphic representation of the randomization process and trial-group assignment, including the pre-specified primary and secondary endpoints.**

AKI, acute kidney injury; CCI, comprehensive complications index; DHOPE, dual hypothermic oxygenated machine perfusion; SADE, serious adverse device event; SAE, serious adverse event; SCS, static cold storage; LT, liver transplantation

**
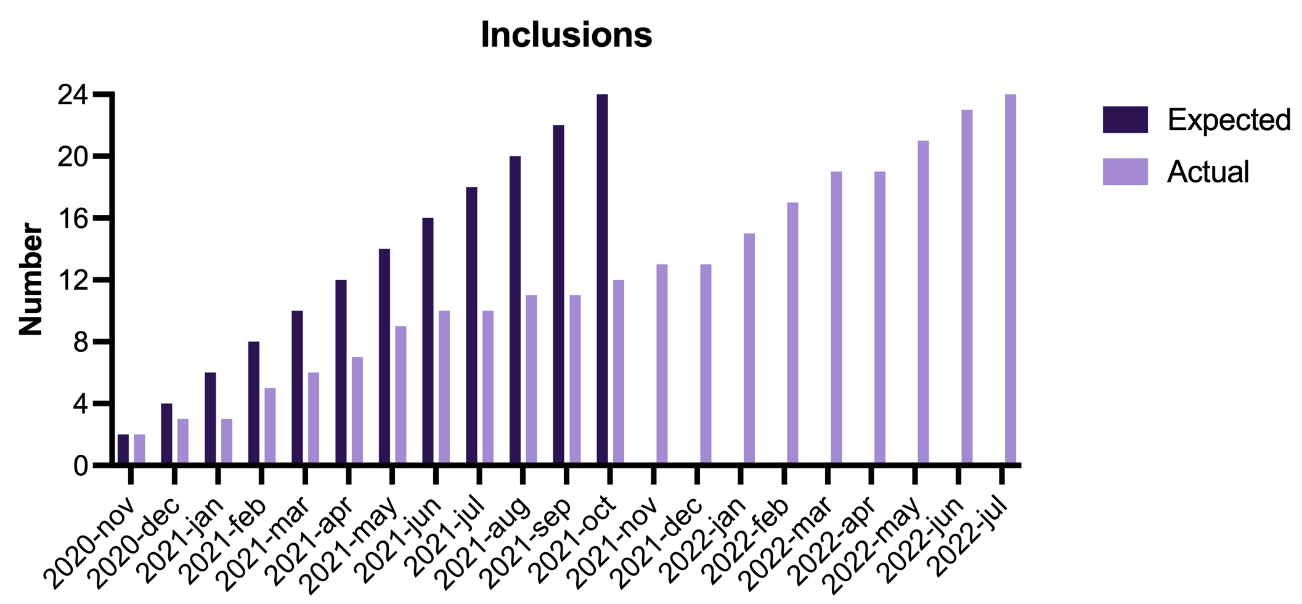
**

**Figure S2. Expected versus actual inclusions during the trial study period.**

**
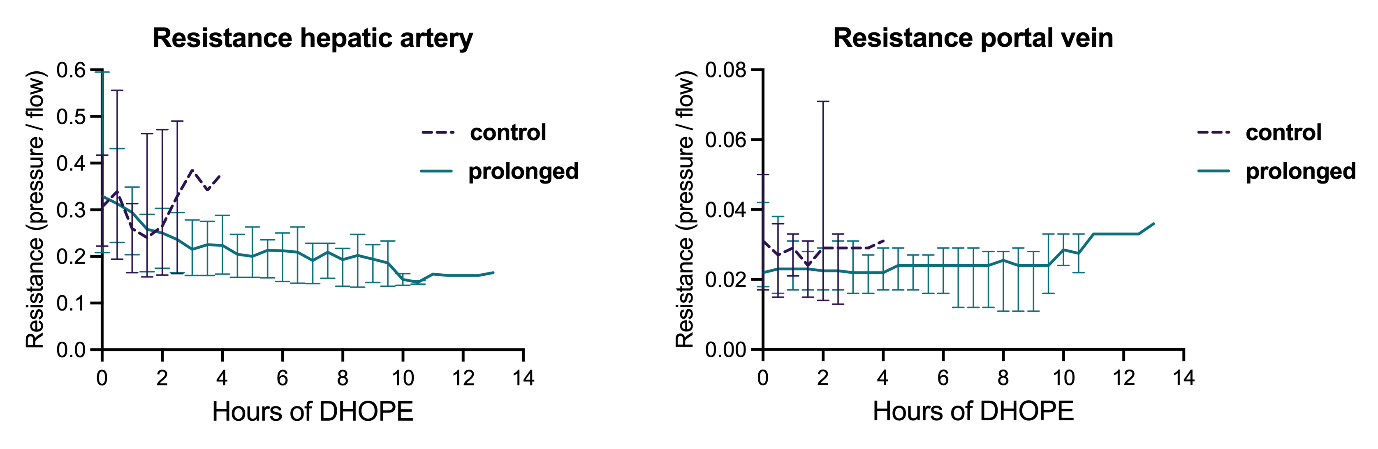
**

**Figure S3. Median perfusion resistance (pressure/flow) of the hepatic artery (HA; left panel) and portal vein (PV; right panel) during dual hypothermic oxygenated machine perfusion (DHOPE) for the control group (n=12), and for the prolonged preservation group (n=12).** The middle line is the median, the lower and upper whisker represent the 95% confidence interval.

**List of DHOPE-PRO Trial Investigators**

The DHOPE-PRO trial was investigator-initiated and designed as a single center, prospective, dual arm, pseudo-randomized, clinical trial. The trial was coordinated and carried out by the University Medical Center Groningen in Groningen, the Netherlands, the trial sponsor.

The following persons participated in the coordination and conduct of the DHOPE-PRO trial:

Coordinating Investigator/Project Leader: Vincent E. de Meijer

Trial Coordinators: Isabel M.A. Brüggenwirth and Veerle A. Lantinga

Writing Committee: Isabel M.A. Brüggenwirth, Veerle A. Lantinga, Vincent E. de Meijer

Data and Safety Monitoring Board: Cyril Moers, Diethard Monbaliu, Sijbrand H. Hofker, Jan Bottema

Trial Monitor: Hildegaard S. Franke

Full list of trial site contributors to the study for PubMed indexation: see list below.

| **First name** | **Surname** | **Affiliation** |
| --- | --- | --- |
| Marieke T. | de Boer | Department of Surgery, University Medical Center Groningen, Groningen, the Netherlands |
| Anne Loes | van den Boom | Department of Surgery, University Medical Center Groningen, Groningen, the Netherlands |
| Carlijn I. | Buis | Department of Surgery, University Medical Center Groningen, Groningen, the Netherlands |
| Suomi M.G. | Fouraschen | Department of Surgery, University Medical Center Groningen, Groningen, the Netherlands |
| Frederik J.H. | Hoogwater | Department of Surgery, University Medical Center Groningen, Groningen, the Netherlands |
| Vincent E. | de Meijer | Department of Surgery, University Medical Center Groningen, Groningen, the Netherlands |
| Joost M. | Klaase | Department of Surgery, University Medical Center Groningen, Groningen, the Netherlands |
| Ruben H.J. | de Kleine | Department of Surgery, University Medical Center Groningen, Groningen, the Netherlands |
| Mark | Meerdink | Department of Surgery, University Medical Center Groningen, Groningen, the Netherlands |
| Maarten W. | Nijkamp | Department of Surgery, University Medical Center Groningen, Groningen, the Netherlands |
| Robert J. | Porte | Department of Surgery, University Medical Center Groningen, Groningen, the Netherlands |
| A. Michel | Rayar | Department of Surgery, University Medical Center Groningen, Groningen, the Netherlands |
| Aad P. | van den Berg | Department of Gastroenterology, University Medical Center Groningen, Groningen, the Netherlands |
| Hans | Blokzijl | Department of Gastroenterology, University Medical Center Groningen, Groningen, the Netherlands |
| Frans J.C. | Cuperus | Department of Gastroenterology, University Medical Center Groningen, Groningen, the Netherlands |
| Frans | van der Heide | Department of Gastroenterology, University Medical Center Groningen, Groningen, the Netherlands |
| Frederike G.I. | van Vilsteren | Department of Gastroenterology, University Medical Center Groningen, Groningen, the Netherlands |
| Ilhama F. | Abbasova | Department of Anesthesiology, University Medical Center Groningen, Groningen, the Netherlands |
| Meine H. | Fernhout | Department of Anesthesiology, University Medical Center Groningen, Groningen, the Netherlands |
| Peter | Meyer | Department of Anesthesiology, University Medical Center Groningen, Groningen, the Netherlands |
| Ernesto R.R. | Muskiet | Department of Anesthesiology, University Medical Center Groningen, Groningen, the Netherlands |
| Koen M.E.M. | Reyntjens | Department of Anesthesiology, University Medical Center Groningen, Groningen, the Netherlands |
| Jaap J. | Vos | Department of Anesthesiology, University Medical Center Groningen, Groningen, the Netherlands |
| Miriam | Zeillemaker | Department of Anesthesiology, University Medical Center Groningen, Groningen, the Netherlands |
| Isabel M.A. | Brüggenwirth | Department of Surgery, University Medical Center Groningen, Groningen, the Netherlands |
| Martijn P.D. | Haring | Department of Surgery, University Medical Center Groningen, Groningen, the Netherlands |
| Veerle A. | Lantinga | Department of Surgery, University Medical Center Groningen, Groningen, the Netherlands |
| Bianca | Lascaris | Department of Surgery, University Medical Center Groningen, Groningen, the Netherlands |
| Carol C. | Pamplona | Department of Surgery, University Medical Center Groningen, Groningen, the Netherlands |
| Adam M. | Thorne | Department of Surgery, University Medical Center Groningen, Groningen, the Netherlands |
| Vivianne | Veenma | Department of Surgery, University Medical Center Groningen, Groningen, the Netherlands |
| Otto B. | van Leeuwen | Department of Surgery, University Medical Center Groningen, Groningen, the Netherlands |
| Silke B. | Bodewes | Department of Surgery, University Medical Center Groningen, Groningen, the Netherlands |
| Ton | Lisman | Department of Surgery, University Medical Center Groningen, Groningen, the Netherlands |
| Jelle | Adelmeijer | Department of Surgery, University Medical Center Groningen, Groningen, the Netherlands |
| Janneke | Wiersema-Buist | Department of Surgery, University Medical Center Groningen, Groningen, the Netherlands |
| Marius | van den Heuvel | Department of Pathology, University Medical Center Groningen, Groningen, the Netherlands |
| Cyril | Moers | Department of Surgery, University Medical Center Groningen, Groningen, the Netherlands |
| Diethard | Monbaliu | Department of Abdominal Transplantation Surgery, University Hospitals KU Leuven, Belgium |
| Sijbrand H. | Hofker | Department of Surgery, University Medical Center Groningen, Groningen, the Netherlands |
